# Efficacy and safety of baricitinib in Japanese patients with autoinflammatory type I interferonopathies (NNS/CANDLE, SAVI, And AGS)

**DOI:** 10.1186/s12969-023-00817-8

**Published:** 2023-04-22

**Authors:** Nobuo Kanazawa, Taeko Ishii, Yasushi Takita, Atsushi Nishikawa, Ryuta Nishikomori

**Affiliations:** 1 Department of Dermatology, Hyogo Medical University, Nishinomiya, Japan; 2 Eli Lilly Japan K.K, Lilly Plaza One Bldg., 5-1-28, Isogamidori, Chuo-ku, Kobe, 651-0086 Japan; 3 Department of Pediatrics and Child Health, Kurume University School of Medicine, Kurume, Japan

**Keywords:** AGS, Autoinflammatory type I interferonopathies, Baricitinib, NNS/CANDLE, SAVI

## Abstract

**Background:**

This study evaluated the efficacy and safety of baricitinib (Janus kinase-1/2 inhibitor), in adult and pediatric Japanese patients with Nakajo-Nishimura syndrome/chronic atypical neutrophilic dermatosis with lipodystrophy and elevated temperature (NNS/CANDLE), stimulator of interferon genes-associated vasculopathy with onset during infancy (SAVI), or Aicardi-Goutières syndrome (AGS).

**Methods:**

A Phase 2/3, multicenter, open-label study (NCT04517253) was conducted across 52 weeks. Primary efficacy endpoint assessed the change in mean daily diary score (DDS) from baseline to the end of primary treatment period. Other efficacy endpoints included change in mean DDS to the end of maintenance period, daily corticosteroid use, Physician’s Global Assessment of Disease Activity (PGA) scores, and daily symptom-specific score (DSSS) from baseline to primary and maintenance treatment periods. All treatment-emergent adverse events (TEAEs) that occurred postdosing were recorded.

**Results:**

Overall, 9 patients (5 with NNS, 3 with SAVI, and 1 with AGS) were enrolled; 55.6% were females, mean age was 26 years, and mean corticosteroid use/weight was 0.2 mg/kg. At the end of primary treatment period, mean DDS decreased from baseline in patients with NNS/CANDLE (0.22) and SAVI (0.21) and increased in the patient with AGS (0.07). At the end of maintenance treatment period, mean DDS decreased from baseline in patients with NNS/CANDLE (0.18) and SAVI (0.27) and increased in the patient with AGS (0.04). Mean percent corticosteroid use decreased by 18.4% in 3 out of 5 patients with NNS/CANDLE and 62.9% in 1 out of 3 patients with SAVI. Mean PGA score decreased from baseline in patients with NNS/CANDLE (1.60), SAVI (1.33), and AGS (1.0), and mean DSSS improved from baseline. All patients reported ≥ 1 TEAE. Frequently reported AEs included BK polyomavirus detection (3; 33.3%), increased blood creatine phosphokinase (2; 22.2%), anemia (2; 22.2%), and upper respiratory tract infection (2; 22.2%). Three (33.3%) patients reported serious adverse events, 1 of which was related to study drug. One patient with SAVI died due to intracranial hemorrhage, which was not related to study drug.

**Conclusion:**

Baricitinib may offer a potential therapeutic option for patients with NNS/CANDLE, SAVI, and AGS, with a positive benefit/risk profile in a vulnerable patient population with multiple comorbidities.

**Trial registration:**

NLM clinicaltrials.gov, NCT04517253. Registered 18 August 2020.

**Supplementary Information:**

The online version contains supplementary material available at 10.1186/s12969-023-00817-8.

## Background

Chronic atypical neutrophilic dermatosis with lipodystrophy and elevated temperature (CANDLE), stimulator of interferon genes (STING)-associated vasculopathy with onset during infancy (SAVI), and Aicardi-Goutières syndrome (AGS) are Mendelian autoinflammatory interferonopathies characterized by early-onset systemic and organ-specific inflammation and a prominent interferon (IFN) response gene signature (IGS) [1].

CANDLE, SAVI, and AGS are monogenic disorders caused by pathogenic genetic defects [1, 2]. Autosomal recessive loss-of-function (LOF) mutations in the proteasome subunit beta type 8 (*PSMB8*) cause Nakajo-Nishimura syndrome (NNS) with nodular erythema, elongated and thickened fingers, and emaciation [1, 3, 4], and CANDLE [1, 4, 5]. Additionally, LOF mutations in other proteasome subunits (*PSMB9, PSMB4, PSMA3*) and the proteasome assembly proteins could contribute to the disease condition [1]. Clinical manifestations commonly associated with CANDLE include fever, nodular or plaque-like violaceous skin rashes, myositis, and joint contractures [1, 5, 6]. SAVI is caused by dominant gain-of-function mutations in *TMEM173* encoding the STING protein [1]. Clinical manifestations commonly associated with SAVI include rash with fever, vasculopathic lesions in cold sensitive acral areas, paratracheal adenopathy, abnormal pulmonary function tests, myositis, and arthritis [1, 7, 8]. Although AGS is a monogenic disorder, it is genetically heterogenous and caused by mutations in seven genes encoding nucleic-acid-processing enzymes and cytosolic nucleic acid sensor. AGS is characterized by unexplained fevers, hepatosplenomegaly, encephalopathy and white matter disease, “chilblains” or cold-induced acral dermatosis, systemic and pulmonary hypertension, and early-onset, monophasic, congenital infection-like syndrome [1, 2, 9, 10]. Moreover, the cerebrospinal fluid shows chronic lymphocytosis and increased IFN-α levels [10].

Considering the dysregulation of IFNs in CANDLE, SAVI, and AGS, an anti-IFN approach could be a possible therapeutic option. Moreover, due to the presence of a high IGS, patients with CANDLE and SAVI respond poorly to disease-modifying antirheumatic drugs and interleukin-1-blocking agents [11]. In patients with AGS, neurological improvement was variable when administered with corticosteroids such as prednisolone [12]. Janus kinase (JAK)–signal transducer and activator of transcription (STAT) pathway is the principal signal transduction pathway for IFNs [13]. Thus, blocking molecules involved in the JAK–STAT pathway could be a possible therapeutic option for NNS/CANDLE, SAVI, and AGS.

Baricitinib is a selective JAK1/2 inhibitor that reduces the phosphorylation and activation of STATs, consequently modulating inflammation, cellular activation, and proliferation of key immune cells [14]. In Japan, baricitinib is approved for the treatment of rheumatoid arthritis (RA) in patients who have had an inadequate response to conventional treatments (including the prevention of structural joint damage), in patients with moderate to severe atopic dermatitis in patients who have had an inadequate response to topical treatments, alopecia areata for extensive and intractable cases, and pneumonia associated with COVID-19 infection (limited to patients requiring supplemental oxygen) [15]. A population pharmacokinetic (PK) modeling was performed to characterize the PK profile of baricitinib in patients with rare Mendelian autoinflammatory interferonopathies enrolled in the FDA-approved compassionate use program (NCT01724580) [16]. The results indicated a dose-dependent decrease of IFN biomarkers and IGS, confirming an in vivo effect of baricitinib and supporting a weight- and estimated glomerular filtration rate (eGFR)-based baricitinib dosing regimen as a therapeutic option for patients with Mendelian autoinflammatory interferonopathies [16].

This study evaluates the efficacy and safety of baricitinib in pediatric and adult Japanese patients with NNS/CANDLE, SAVI, and AGS. The safety and tolerability data from this study are intended to establish an understanding of the benefit/risk relationship of baricitinib in patients with NNS/CANDLE, SAVI, and AGS.

## Methods

### Study population

Patients demonstrating ≥ 2 signs and symptoms and with a confirmed genetic diagnosis of NNS/CANDLE, SAVI, or AGS (including familial chilblain lupus) were eligible. Patients with NNS/CANDLE or SAVI ≥ 17.5 months of age and patients with AGS ≥ 6 months of age were included in the trial [17]. All the patients weighed ≥ 5 kg with average daily dairy score (DDS) of

-  ≥ 0.5 for patients with NNS/CANDLE at Visit 2,
-  ≥ 1.0 for patients with SAVI at Visit 6, and
-  ≥ 0.5 for patients with AGS at Visit 6

### Study design

This Phase 2/3, multicenter, open-label study (ClinicalTrials.gov Identifier: NCT04517253) evaluated the efficacy and safety of baricitinib in adult and pediatric Japanese patients with autoinflammatory type I interferonopathies including NNS/CANDLE, SAVI, and AGS.

**Fig. 1:**
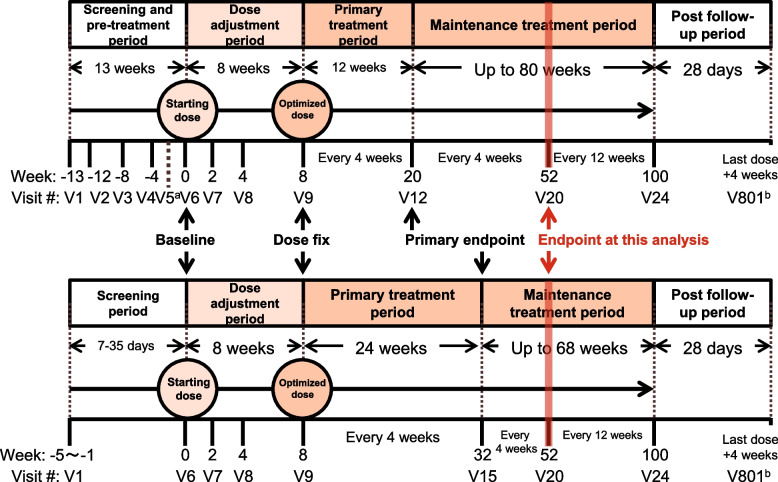
Study design. AGS = Aicardi-Goutières syndrome; NNS/CANDLE = Nakajo-Nishimura syndrome/chronic atypical neutrophilic dermatosis with lipodystrophy and elevated temperature; SAVI = STING-associated vasculopathy with onset during infancy; STING = stimulator of interferon genes; V = visit; wk = week. ^a^Patient can skip Visit 5 and proceed to Visit 6 if biologic agents are not administered during pre-treatment period or an appropriate washout duration of biologic agents defined in Exclusion Criterion #28 has already passed at Visit 5. ^b^Visit 801 should occur approximately 28 days after the last dose of study drug. Patients who will transition from this study to commercial baricitinib are not required to complete Visit 801

The study was divided into 6 periods including (i) screening, (ii) pre-treatment (only for patients with NNS/CANDLE), (iii) dose adjustment, (iv) primary treatment, (v) maintenance treatment, and (vi) post-treatment follow-up period (Fig. 1).

The screening period varied for patients with NNS/CANDLE, SAVI, and AGS. The screening period ranged from 7 to 35 days prior to baseline (Visit 6) for patients with SAVI and AGS, whereas patients with NNS/CANDLE had a screening period of 1 week (Visit 1) before entering the pre-treatment period.

Patients with NNS/CANDLE entered a 12-week pre-treatment period at the beginning of Visit 1. The data from the pre-treatment period was used for baseline comparison.

Patients who met all eligibility criteria entered the 8-week dose adjustment period (Visit 6 to Visit 9). Patients initially received treatment dose based on weight class and eGFR, which was further escalated to identify tolerable dose. The dose escalation model was based on the results from previous PK studies of baricitinib [11, 16]. Patients who weighed < 40 kg received baricitinib tablets or liquid suspension orally based on patient choice, whereas patients who weighed ≥ 40 kg were recommended to receive only tablets. If the patients’ weight changed during the study, dose regimens could be modified. Compliance was assessed by counting returned tablets or weighing a returned bottle for liquid suspension. Patients treated with baricitinib were considered noncompliant if they missed ≥ 20% of the prescribed doses during the study or if they were judged by the investigator to have intentionally or repeatedly taken more than the prescribed amount of study medication.

Patients received the optimized dosage throughout the primary and maintenance treatment periods. The duration of primary treatment period was 12 weeks for patients with NNS/CANDLE and 24 weeks for patients with SAVI and AGS based on the results from the JAGA (NCT01724580) trial [11]. Entry into the maintenance period after completion of the primary treatment period occurred at Visit 12 for patients with NNS/CANDLE and Visit 15 for patients with SAVI and AGS. This study is ongoing to collect long-term efficacy and safety results.

Treatment period was considered from the first dose of baricitinib (Visit 6; Week 0) to the date of final visit or early discontinuation period. Endpoint of post-follow-up period was Visit 801. Primary endpoint assessed the change in mean DDS from baseline (Week 0; Visit 6) to Week 20 in patients with NNS/CANDLE and Week 32 in patients with SAVI and AGS. Other efficacy and safety endpoints were assessed until Week 52.

The study protocol was approved by the institutional review boards prior to patient recruitment, and each patient or their guardian provided written informed consent prior to enrollment. The study was conducted in accordance with consensus ethics principles derived from international ethics guidelines, including the Declaration of Helsinki and the International Ethical Guidelines by the Council for International Organizations of Medical Sciences and the International Council for Harmonization E6 Guidelines for Good Clinical Practice.

### Clinical benefit assessment

#### Decrease in daily diary score

The primary effectiveness measure evaluated the decrease in DDS of patients’ signs and symptoms. Daily symptoms, including fever, rash, musculoskeletal pain, headache, and fatigue, were rated with increasing level of severity. Signs and symptoms for NNS/CANDLE, SAVI, and AGS are detailed in Supplementary Table 1. The average score of each symptom was calculated using the data from 7 days preceding the current visit. The calculated average score for each symptom is summed up and divided by the number of assessed symptoms (5 symptoms for NNS/CANDLE, 6 symptoms for SAVI, and 8 symptoms for AGS) to calculate the average score for each patient. DDS does not change linearly; therefore, no settings for "minimal clinically important difference” could be set. However, patients were considered well-controlled if a DDS of < 0.5 for NNS/CANDLE and < 1.0 for SAVI was observed [11].

#### Decrease in daily dose of corticosteroids

A decrease in daily dose of corticosteroids was defined by a < 0.15 mg/kg/day prednisone equivalent systemic corticosteroid dose or a daily dose percentage decrease of at least 50% from baseline.

#### Decrease in physician’s global assessment of disease activity score

The patients’ current disease activities were assessed using the 21-circle visual analog scale ranging from 0 to 10 with increasing level of severity [18].

#### Other clinical assessments

An improvement in diary symptom-specific score (DSSS) from baseline, Barthel index evaluating the activity of living with intractable diseases [19], mean changes in height and growth from baseline, and laboratory parameters including C-reactive protein (CRP), aspartate transaminase, alanine aminotransferase, gamma glutamyl transferase, and creatine phosphokinase were assessed.

Mean change from baseline in biomarkers of IFN signaling and IGS was assessed throughout the study. The IFN signature score used in IGS assessment, comprised of 6-gene IFN signature assays from 28 IFN Response Genes. The final assay was parameterized to separate IFN-high and IFN-low patients with a cut point of zero where each gene was scaled to have the same range. A change of 1 point in the IFN signature means twofold change in gene concentration.

#### Safety assessment

All adverse events (AEs) occurring after signing the informed consent form were recorded in the electronic case report form. AEs were classified based on the Medical Dictionary for Regulatory Activities. AE of special interest included infections, myelosuppressive events, thrombocytosis, malignancies, hepatic events, major adverse cardiovascular events, and thrombotic events. Electrocardiograms, physical examinations, vital signs including lung and liver function tests, BK virus plasma and urine screening using quantitative PCR, hepatitis B virus DNA monitoring (only in patients who were positive for HbcAb or HbsAb at Visit 1), and height and weight measurement were performed, and clinically significant findings were reported.

#### Statistics

In lieu of anticipating that relatively few patients with each condition would be enrolled, no formal statistical tests were planned. Instead, descriptive summaries and data listings were planned to summarize the results.

Continuous data were summarized in terms of the mean, standard deviation (SD), minimum, maximum, median, and number of observations; categorical data were summarized as frequency counts and percentages. Although the efficacy population set included all enrolled patients who had at least 1 dose of baricitinib, efficacy analyses were divided among diagnosis-specific efficacy population subsets.

Mean DDS was calculated from the average scores of 7 days preceding the current visit; however, if more than 50% symptom scores were missing, the mean DDS was not calculated. A last-observation-carried-forward imputation replaced missing data with the most recent non-missing postbaseline assessment. Proportion of days meeting threshold was calculated by the total number of days meeting the threshold divided by total number of days with non-missing diary scores during the interval. Mean DDS with < 0.5 was used as a threshold for all diseases as an indication of disease control. All corticosteroid doses were standardized to an equivalent prednisone dose.

## Results

### Study population

A total of 9 patients were enrolled in the study. Five patients were diagnosed with NNS/CANDLE, 3 patients with SAVI, and 1 patient with AGS. One patient with NNS/CANDLE discontinued the study treatment and study due to an AE and one patient with SAVI discontinued the study treatment and study due to death.

Overall, the mean age (SD) of patients was 26 (19.1) years, a higher proportion were female (5; 55.6%), and the mean baseline eGFR was 117.6 mL/min/1.73 m^2^ (Table 1). All patients had a history of concomitant medications, with a majority of patients using prednisolone (8; 88.9%), and the mean corticosteroid dose per weight was 0.2 mg/kg (Table 1).

**Table 1:** Summary of demographic and baseline characteristics

Parameter	NNS/CANDLE (*N* = 5)	SAVI (*N* = 3)	AGS (*N* = 1)	Total (*N* = 9)
Age, years				
Mean (SD)	39.2 (13.8)	9.7 (9.8)	9.0 (NA)	26.0 (19.1)
Range (minimum, maximum)	15–48	4–21	9–9	4–48
Sex, n (%)				
Female	1 (20.0)	3 (100.0)	1 (100.0)	5 (55.6)
eGFR (mL/min/1.73m^2^)				
Mean (SD)	124.7 (19.3)	109.4 (22.5)	106.7 (NA)	117.6 (19.6)
Weight, kg				
Mean (SD)	45.8 (5.9)	22.7 (17.3)	14.30 (NA)	34.6 (16.6)
Weight, n (%)				
≥ 10 to < 20 kg	0	2 (66.7)	1 (100.0)	3 (33.3)
≥ 40 to < 50 kg	4 (80.0)	1 (33.3)	0	5 (55.6)
≥ 50 to < 60 kg	1 (20.0)	0	0	1 (11.1)
Height (cm)				
Mean (SD)	150.9 (11.2)	112.9 (31.2)	112.2 (NA)	133.9 (26.6)
BMI (kg/m^2^)				
Mean (SD)	20.2 (2.3)	15.7 (3.2)	11.4 (NA)	17.7 (4.0)
Corticosteroid use				
n (%)	5 (100.0)	1 (33.3)	0	6 (66.7)
Corticosteroid total daily dose (mg)^a^				
Mean (SD)	8.9 (4.3)	4 (NA)	0	8.08 (4.3)
Corticosteroid dose per weight (mg/kg)^a^				
Mean (SD)	0.2 (0.1)	0.4 (NA)	0	0.2 (0.1)

*AGS* Aicardi-Goutières syndrome, *BMI* body mass index, *eGFR* estimated glomerular filtration rate, *n* number of patients with non-missing data, *N* number of patients in the safety population within each disease subpopulation, *NA* not applicable, *NNS/CANDLE* Nakajo-Nishimura syndrome/chronic atypical neutrophilic dermatosis with lipodystrophy and elevated temperature, *SAVI* STING-associated vasculopathy with onset during infancy, *SD* standard deviation, *STING* stimulator of interferon genes

^a^The mean corticosteroid doses are reported as prednisone equivalent doses in patients taking corticosteroids

### Treatment

The maximum total daily dose of baricitinib ranged from 8 to 12 mg. The median (range) duration of exposure to baricitinib was 52.1 (8.4–53.1) weeks. Among patients with NNS/CANDLE, the minimum and maximum duration of exposure was 13.3 weeks and 52.4 weeks, respectively. In patients with SAVI, the minimum and maximum duration of exposure was 8.4 weeks and 53.0 weeks, respectively; 1 patient discontinued early due to death and 1 patient was enrolled in the study after primary data cutoff and did not complete 52 weeks as of April 2022. In the patient with AGS, the duration of exposure was 53.1 weeks. Patients were deemed compliant if they missed < 20% of the expected number of doses; all patients were compliant in the study.

### Efficacy

#### Primary efficacy endpoint

##### Mean daily diary score at the end of the primary treatment period

The overall mean DDS decreased from baseline in patients with NNS/CANDLE (0.22) and SAVI (0.21), but slightly increased in the patient with AGS (0.07) (Table 2).

**Table 2:** Summary of efficacy parameters by subgroup

			NNS/CANDLE (*N* = 5)		SAVI (*N* = 3)		AGS (*N* = 1)	
Parameters	Period	Week	Observed	Change	Observed	Change	Observed	Change
Mean DDS, mean (SD)	Pre-treatment^a^		0.64 (0.32)		-		-	
	Baseline	0	0.92 (0.35)		0.79 (0.28)		1.25 (NA)	
	Primary treatment^b^	20/32	0.70 (0.51)	-0.22 (0.59)	0.57 (0.23)	-0.21 (0.25)	1.3 (NA)	0.07 (NA)
	Maintenance treatment	52	0.74 (0.57)	-0.18 (0.64)	0.52 (0.28)	-0.27 (0.34)	1.29 (NA)	0.04 (NA)
Proportion of days with < 0.5 in mean DDS, mean (SD)	Pre-treatment^a^		0.36 (0.24)		-		-	
	Baseline	0	0.0 (0)		0.23 (0.39)		0 (NA)	
	Primary treatment^b^	20/32	0.40 (0.55)	0.40 (0.55)	0.32 (0.56)	0.10 (0.17)	0 (NA)	0.0 (NA)
	Maintenance treatment	52	0.36 (0.50)	0.36 (0.50)	0.63 (0.55)	0.41 (0.47)	0 (NA)	0.0 (NA)
Physician’s Global Assessment of disease activity, mean (SD)	Pre-treatment^a^		5.45 (1.33)		-		-	
	Baseline	0	5.80 (1.15)		3.83 (1.26)		2.50 (NA)	
	Primary treatment	20/32	3.70 (1.10)	-2.10 (0.82)	2.50 (1.80)	-1.33 (1.76)	2.0 (NA)	-0.50 (NA)
	Maintenance treatment	52	4.20 (2.80)	-1.60 (1.85)	2.50 (1.80)	-1.33 (1.76)	1.50 (NA)	-1.0 (NA)
Dose of systemic corticosteroid with prednisone equivalent (mg/kg/day)^c^	Baseline, mean (SD)	0	0.19 (0.09)		0.39 (NA)		-	
	Primary treatment, mean (SD)	20/32	0.15 (0.15)	-0.04 (0.10)	0.15 (NA)	-0.24 (NA)	-	-
	Primary treatment, % change			-23.5% (41.5%)		-61.2% (NA)		
	Maintenance treatment, mean (SD)	52	0.17 (0.15)	-0.03 (0.11)	0.14 (NA)	-0.24 (NA)	-	-
	Maintenance treatment, % change			-18.4% (43.4%)		-62.9% (NA)		

*AGS* Aicardi-Goutières syndrome, *DDS* daily diary score, *N* total number of patients, *NA* not applicable, *NNS/CANDLE* Nakajo-Nishimura syndrome/chronic atypical neutrophilic dermatosis with lipodystrophy and elevated temperature, *SAVI* STING-associated vasculopathy with onset during infancy, *STING* stimulator of interferon genes

Patients with SAVI and AGS had no pre-treatment scores

^a^Averaged scores collected during pre-treatment period were used

^b^Week 20 for NNS/CANDLE; Week 32 for SAVI and AGS

^c^Patients on systemic corticosteroid at baseline were included

At the end of the primary treatment period (Week 20), patients with NNS/CANDLE met the response criterion of mean DDS < 0.5 with a mean proportion of days of 0.4 and a change from the pre-treatment baseline with a mean proportion of days of 0.04 (Table 2).

#### Secondary endpoint

##### Mean daily diary score at the end of the maintenance treatment period

**Fig. 2:**
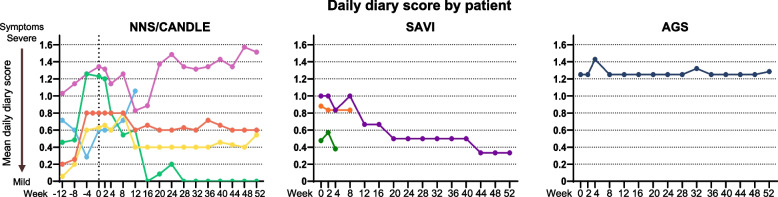
Mean daily diary score by patients with (**A**) NNS/CANDLE, (**B**) SAVI, and (**C**) AGS. AGS = Aicardi-Goutières syndrome; NNS/CANDLE = Nakajo-Nishimura syndrome/chronic atypical neutrophilic dermatosis with lipodystrophy and elevated temperature; SAVI = STING-associated vasculopathy with onset during infancy; STING = stimulator of interferon genes

The overall mean DDS decreased from baseline in patients with NNS/CANDLE (0.18) and SAVI (0.27), but slightly increased for the patient with AGS (0.04) (Table 2 and Fig. 2). Three of 5 patients with NNS/CANDLE and all 3 patients with SAVI showed decreased mean DDS at last observation compared with baseline (Fig. 2).

At the end of the maintenance treatment period (Week 52), patients with NNS/CANDLE met the response criteria of mean DDS < 0.5 with a mean proportion of days of 0.36 and a change from the pre-treatment baseline with a mean proportion of days of 0.01 (Table 2 and Fig. 2).

##### Daily dose of corticosteroid

**Fig. 3:**
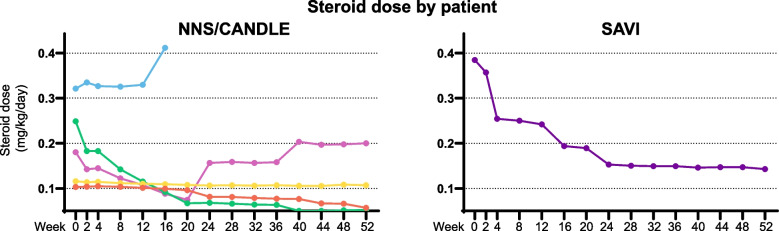
Corticosteroid use in patients with (**A**) NNS/CANDLE and (**B**) SAVI. NNS/CANDLE = Nakajo-Nishimura syndrome/chronic atypical neutrophilic dermatosis with lipodystrophy and elevated temperature; SAVI = STING-associated vasculopathy with onset during infancy; STING = stimulator of interferon genes

At the end of the primary treatment period, all 5 patients with NNS/CANDLE reported corticosteroid use at baseline and showed a mean decrease of 23.5% in systemic corticosteroid use with a mean change of -0.04 mg/kg/day from baseline. One patient with SAVI reported corticosteroid use at baseline and showed a clinically significant mean percent decrease of 61.2% in corticosteroid use and a mean change of -0.24 mg/kg/day from baseline (Table 2 and Fig. 3).

At the end of the maintenance period, patients with NNS/CANDLE showed a mean percent decrease of 18.4% in systemic corticosteroids use with a mean change of -0.03 mg/kg/day from baseline. The patient with SAVI showed a clinically significant mean percent decrease of 62.9% in systemic corticosteroids use and a mean change of -0.24 mg/kg/day from baseline (Table 2 and Fig. 3). The patient with AGS did not use any corticosteroid at baseline.

##### Physician’s global assessment of disease activity score

At the end of the primary treatment period, patients with NNS/CANDLE (2.10), SAVI (1.33), and AGS (0.50) showed mean decrease in Physician’s Global Assessment of Disease Activity (PGA) score from baseline. Decrease in PGA score (1.75) from pre-treatment baseline was observed in patients with NNS/CANDLE (Table 2).

A similar trend was observed at the end of the maintenance period (Week 52). Patients with NNS/CANDLE (1.60), SAVI (1.33), and AGS (1.0) showed mean decrease in PGA score from baseline. Decrease in PGA score (1.25) from pre-treatment baseline was observed in patients with NNS/CANDLE (Table 2).

**Fig. 4:**
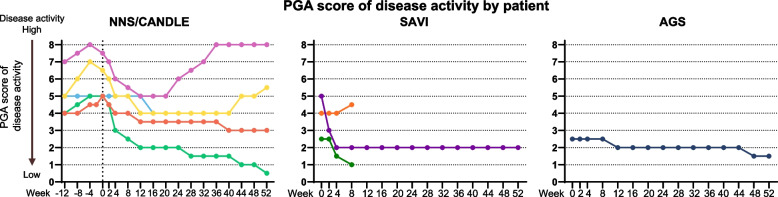
PGA scores in patients with (**A**) NNS/CANDLE, (**B**) SAVI, and (**C**) AGS. AGS = Aicardi-Goutières syndrome; NNS/CANDLE = Nakajo-Nishimura syndrome/chronic atypical neutrophilic dermatosis with lipodystrophy and elevated temperature; SAVI = STING-associated vasculopathy with onset during infancy; STING = stimulator of interferon genes

Individual patient PGA scores are listed in Fig. 4.

##### Other efficacy analyses

DSSS across all patient subgroups improved from baseline. Rash, musculoskeletal pain, and fatigue symptom scores improved in patients with NNS/CANDLE. Fever, rash, musculoskeletal pain, fatigue, and respiratory/breathing symptom scores improved in patients with SAVI, and length of uninterrupted sleep increased in the patient with AGS (Supplementary Table 2).

A mean decrease from baseline in disease severity of fever and rash was observed in patients with NNS/CANDLE. Patients with SAVI and AGS were assessed using the Barthel index, with 1 patient with SAVI demonstrating an increase in index scores for transfers and mobility on level surfaces and stairs.

### Safety

#### Adverse events

All 9 patients across disease subgroups reported at least 1 treatment-emergent adverse event (TEAE). Overall, a higher proportion 4 (44.4%) of patients reported mild TEAEs. At the end of the primary treatment period, 3 (33.3%) patients reported serious adverse events (SAEs) including acute coronary syndrome, pancytopenia, and bronchopulmonary aspergillosis, of which pancytopenia was deemed related to the study drug. One (11.1%) patient with SAVI was reported dead due to intracranial hemorrhage, which was not related to the study drug, and 1 (11.1%) patient with NNS/CANDLE discontinued study treatment due to *Pneumocystis jirovecii* pneumonia, which was related to the study drug (Table 3). No SAEs or deaths were reported during the maintenance treatment period.

**Table 3:** Summary of adverse events during the dose adjustment and primary treatment period, and maintenance period

Incidence, n (%)	NNS/CANDLE (*N* = 5) n (%)	SAVI (*N* = 3) n (%)	AGS (*N* = 1) n (%)	Total (*N* = 9) n (%)
TEAE^a^	5 (100.0)	3 (100.0)	1 (100.0)	9 (100.0)
Severe TEAEs^b^	2 (40.0)	1 (33.3)	0 (0.0)	3 (33.3)
Moderate TEAEs^b^	2 (40.0)	0 (0.0)	0 (0.0)	2 (22.2)
Mild TEAEs^b^	1 (20.0)	2 (66.7)	1 (100.0)	4 (44.4)
Death^c^	0 (0.0)	1 (33.3)^c^	0 (0.0)	1 (11.1)
SAE	2 (40.0)	1 (33.3)^c^	0 (0.0)	3 (33.3)
Discontinuation from study and study treatment due to AE	1 (20.0)	1 (33.3)^c^	0 (0.0)	2 (22.2)

*AE* adverse event, *AGS* Aicardi-Goutières Syndrome, *n* number of patients with at least one adverse event per event type, *NNS/CANDLE* Nakajo-Nishimura syndrome/chronic atypical neutrophilic dermatosis with lipodystrophy and elevated temperature, *SAE* serious adverse event, *SAVI* STING-associated vasculopathy with onset during infancy, *STING* stimulator of interferon genes, *TEAE* treatment-emergent adverse event

^a^Patients may be counted in more than one category

^b^Patients with multiple occurrences of the same event are counted under the highest severity

^c^The death event was included in SAE and discontinuations due to AE

The most frequently reported events by preferred term included BK polyomavirus test positive (3; 33.3%), increased blood creatine phosphokinase (2; 22.2%), anemia (2; 22.2%), and upper respiratory tract infection (2; 22.2%).

#### Adverse events of special interest

A majority (6; 66.7%) of patients had infections. Upper respiratory tract infections were observed in 2 patients (22.2%). All other infections including BK virus infection were observed in 1 patient each, across NNS/CANDLE, SAVI, and AGS groups (Table 4).

**Table 4:** Adverse events of special interest – infections

Preferred Term	NNS/CANDLE (*N* = 5) n (%)	SAVI (*N* = 3) n (%)	AGS (*N* = 1) n (%)	Total (*N* = 9) n (%)
Patients ≥ 1 TEAE	3 (60.0)	2 (66.7)	1 (100.0)	6 (66.7)
Upper respiratory tract infection	1 (20.0)	1 (33.3)	0 (0.0)	2 (22.2)
Atypical mycobacterial infection	0	1 (33.3)	0 (0.0)	1 (11.1)
BK virus infection	1 (20.0)	0	0 (0.0)	1 (11.1)
Bronchopulmonary aspergillosis	0 (0.0)	1 (33.3)	0 (0.0)	1 (11.1)
Conjunctivitis	0 (0.0)	1 (33.3)	0 (0.0)	1 (11.1)
Cytomegalovirus chorioretinitis	1 (20.0)	0 (0.0)	0 (0.0)	1 (11.1)
Folliculitis	1 (20.0)	0 (0.0)	0 (0.0)	1 (11.1)
Localized infection	1 (20.0)	0 (0.0)	0 (0.0)	1 (11.1)
Periodontitis	1 (20.0)	0 (0.0)	0 (0.0)	1 (11.1)
Pharyngitis	0 (0.0)	0 (0.0)	1 (100.0)	1 (11.1)
Pneumocystis jirovecii pneumonia	1 (20.0)	0 (0.0)	0 (0.0)	1 (11.1)
Pneumonia cytomegaloviral	1 (20.0)	0 (0.0)	0 (0.0)	1 (11.1)
Tinea capitis	1 (20.0)	0 (0.0)	0 (0.0)	1 (11.1)

*AGS* Aicardi-Goutières syndrome, *n* number of patients with at least one adverse event per event type, *NNS/CANDLE* Nakajo-Nishimura syndrome/chronic atypical neutrophilic dermatosis with lipodystrophy and elevated temperature, *SAVI* STING-associated vasculopathy with onset during infancy, *STING* stimulator of interferon genes, *TEAE* treatment-emergent adverse event

#### Clinical laboratory evaluation

No clinically meaningful changes were observed in systolic blood pressure, diastolic blood pressure, or pulse rate.

Mean increase in growth (height and weight) and growth velocity were observed for all 4 patients younger than 18 years of age during the study, from baseline (Visit 6).

#### Biomarkers

**Fig. 5:**
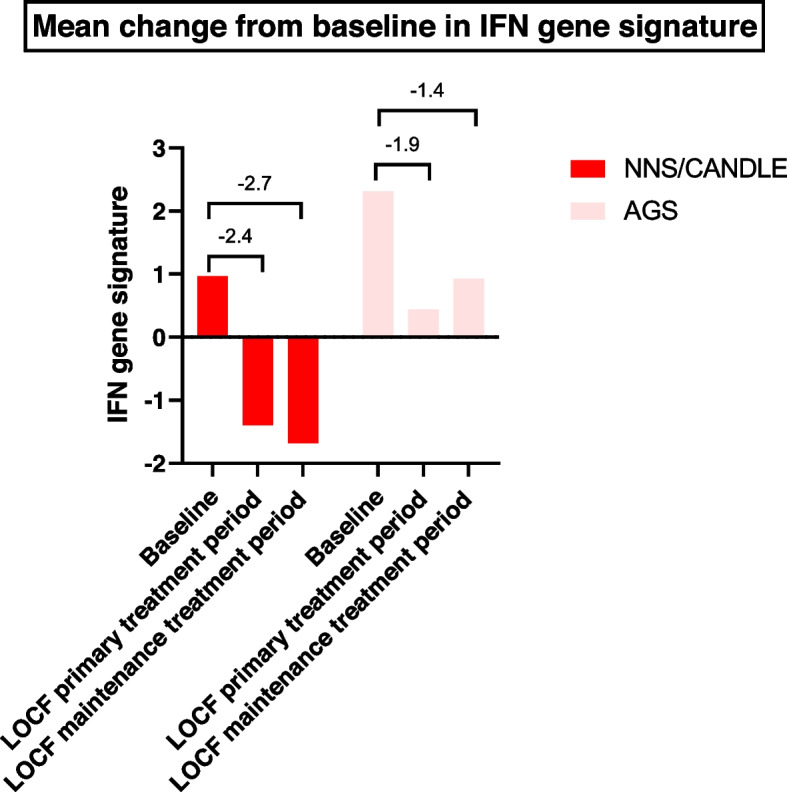
Mean change from baseline in IFN gene score across patients with NNS/CANDLE and AGS. AGS = Aicardi-Goutières syndrome; IFN = interferon; LOCF = last observation carried forward; NNS/CANDLE = Nakajo-Nishimura syndrome/chronic atypical neutrophilic dermatosis with lipodystrophy and elevated temperature; SAVI = STING-associated vasculopathy with onset during infancy; SLE = Systemic Lupus Erythematosus; STING = stimulator of interferon genes. Note: Genes including *OAS3*, *IFI44*, *MX1*, *USP18*, *LY6E*, and *DDX60* were used to calculate the IFN gene signature. The IFN signature score is composed of a 6-gene IFN signature assays from 28 IFN Response Genes of the ModaPlex platform. A 6-gene IFN signature assay was developed by Lilly based on data from more than 2000 SLE patients. The final ModaPlex assay is parameterized to separate IFN-high and IFN-low patients with a cut point of 0 where each gene is scaled to have the same range. A difference of 1 point in the IFN signature correlates with twice the difference in gene concentration. LOCF data were used for imputation. For patients with SAVI, samples were not obtained due to young age; therefore, no patients had both baseline and postbaseline measurements of IFN gene signature

**Fig. 6:**
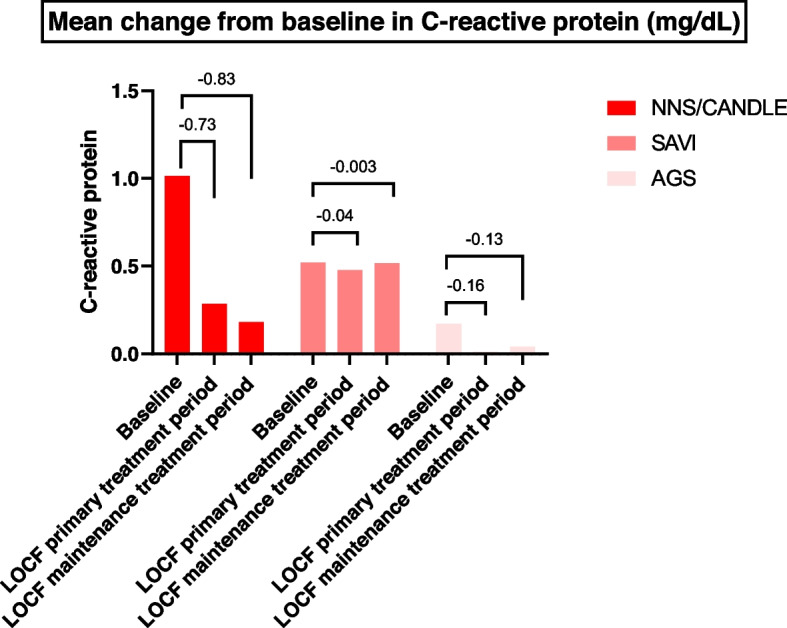
Mean change from baseline in C-reactive protein (mg/dL) across patients with NNS/CANDLE, SAVI, or AGS. AGS = Aicardi-Goutières syndrome; IFN = interferon; LOCF = last observation carried forward; NNS/CANDLE = Nakajo-Nishimura syndrome/chronic atypical neutrophilic dermatosis with lipodystrophy and elevated temperature; SAVI = STING-associated vasculopathy with onset during infancy; STING = stimulator of interferon genes. LOCF data were used for imputation

Interferon-inducible protein 10/C-X-C motif chemokine 10 (IP-10/CXCL10) decreased from baseline in patients with NNS/CANDLE at the end of the primary treatment period (9146.5) and maintenance treatment period (7652.2). Results of IP-10/CXCL10 could not be determined for patients with SAVI and AGS due to limited blood samples. IGS decreased in patients with NNS/CANDLE and AGS consistently throughout the study, with the mean change from baseline at -2.7 and -1.4 at the end of the maintenance period (Week 52), respectively (Fig. 5). CRP level decreased from baseline in patients with NNS/CANDLE (0.83) and in the patient with AGS (0.13) and minor change in patients with SAVI (-0.003) was observed (Fig. 6).

## Discussion

Our study was the first that evaluated the efficacy and safety of baricitinib in Japanese patients with autoinflammatory interferonopathies, NNS/CANDLE, SAVI, or AGS. The overall reduction in mean DDS from baseline in patients with NNS/CANDLE or SAVI at the end of the primary treatment period and maintenance period demonstrated consistent efficacy. Decrease in PGA score and DSSS, consequently leading to decrease in corticosteroid use, reinforced the efficacy of baricitinib in patients with these rare autoinflammatory type I interferonopathies. Four (44.4%) of 9 patients had mild TEAEs, and infections were the most frequently reported. SAEs observed at the end of the primary treatment period were not reported at the end of the maintenance period, and 1 SAE was deemed related to the study drug. Two patients with NNS/CANDLE and SAVI discontinued study due to AE and death, respectively. The patient with SAVI had SAEs, including pneumonia due to aspergillus infection and nontuberculous mycobacterium infection, who consequently died. As per the study investigator, the SAEs were related to the study drug, as the drug was immunosuppressive. However, the death due to intracranial hemorrhage was not deemed to be related to the study drug, as the study drug was discontinued three months prior due to *Pneumocystis jirovecii* pneumonia. The safety results were comparable to an earlier baricitinib trial (NCT01724580) [11]. Mean increases in height and weight was observed in patients younger than 18 years, indicating that this JAK inhibitor potentially does not inhibit growth hormone receptor-induced tyrosine kinase JAK2 phosphorylation [20]. Moreover, biomarkers including IP-10/CXCL10, CRP, and IGS decreased in patients with NNS/CANDLE, indicating a pronounced clinical response.

The results of our study were comparable to the baricitinib trial conducted as an expanded access program in patients with type I IFN-mediated autoinflammatory diseases (JAGA trial; NCT01724580) [11]. Among the individual subgroups of patients, percent improvement in DDS in our study was observed in a lower percent of patients with NNS/CANDLE (60% vs 80%) and higher percent of patients with SAVI (100% vs 75%) compared with the JAGA trial [11]. The variation in the percentage of patients with NNS/CANDLE could be attributed to the mean age difference in the population. In the JAGA trial, the mean age was 12.5 years (range: 1.2–24.1 years), whereas in our study the mean age for patients with NNS/CANDLE was 39.2 years (range: 15–48 years). In concordance with the JAGA trial [11], symptoms and corticosteroid usage decreased across all patient disease subgroups, with the clinical responses being most distinct in patients with NNS/CANDLE. The safety profile of baricitinib in patients with RA [21] was similar to our study, with upper respiratory tract infection being commonly observed. Based on the wide age group, it can be hypothesized that the pediatric population with shorter disease history could respond effectively to baricitinib. Further studies in this population could be helpful.

Our data showed a smaller proportion of patients with upper respiratory tract infections (22.2% vs 77.8%) and BK polyomavirus infection (33.3% vs 50%) than the JAGA trial [11]. This could be due to the smaller sample size in our study, the varied demographics, ethnicity, and duration of the trial. However, both studies indicated that infections were the most frequently reported, and overall, the drug was well tolerated.

BK polyomavirus positivity in patients treated with baricitinib was consistent between our study and the JAGA trial. Safety was assessed and BK viral load was monitored in 7 additional patients after the JAGA trial. Of the total 23 patients in the trial, 20 (87%) developed BK viruria [22]. Observation of BK viruria and viraemia is unique and monitoring an evaluation of BK viral load in patients on chronic immunosuppressive therapy should be considered.

The study had limitations including small sample size, heterogenous patient population, absence of comparators and randomization, use of immunosuppressive concomitant medications, and presence of clinically significant preexisting conditions, leading to potential bias. However, although the enrolled Japanese patient population was small, open label baricitinib consistently decreased mean DDS across 52 weeks. Overall, the drug was well tolerated, but physicians need to be cautious of BK polyomavirus. Continuation of this trial would further provide clarity on the long-term effect and benefit/risk ratio of baricitinib in patients with NNS/CANDLE, SAVI, or AGS.

## Conclusion

In conclusion, the findings suggest that baricitinib could be a potential therapeutic agent with a positive benefit/risk profile in a vulnerable patient population with autoinflammatory interferonopathies.

## Supplementary Information

Additional file 1: Supplementary Table S1. Daily symptoms of patients with NNS/CANDLE, SAVI, and AGS. Supplementary Table S2. Symptom-specific change from baseline in patients with NNS/CANDLE, SAVI, and AGS.

## Data Availability

Eli Lilly and Company provides access to all individual participant data collected during the trial, after anonymization, with the exception of PK or genetic data. Data are available to be requested for 6 months after the indication studied has been approved in the United States and the European Union and after primary publication acceptance, whichever is later. No expiration date of data requests is currently set once data are made available. Access is provided after a proposal has been approved by an independent review committee identified for this purpose and after receipt of a signed data sharing agreement. Data and documents, including the study protocol, statistical analysis plan, clinical study report, and blank or annotated case report forms, will be provided in a secure data-sharing environment. For details on submitting a request, see the instructions provided at https://vivli.org/.

## Abbreviations

AE: Adverse event
AGS: Aicardi-Goutières syndrome
CANDLE: Chronic atypical neutrophilic dermatosis with lipodystrophy and elevated temperature
CRP: C-reactive protein
CXCL10: C-X-C motif chemokine 10
DDS: Daily diary score
DSSS: Daily symptom-specific score
eGFR: Estimated glomerular filtration rate
IGS: Interferon gene signature
IFN: Interferon
IP-10: Interferon-inducible protein 10
JAGA: I4V-MC-JAGA (NCT01724580)
JAK: Janus kinase
LOF: Loss of function
NNS: Nakajo-Nishimura syndrome
PGA: Physician’s Global Assessment of Disease Activity
PK: Pharmacokinetic
RA: Rheumatoid arthritis
SAE: Serious adverse event
SAVI: Stimulator of interferon genes-associated vasculopathy with onset during infancy
SD: Standard deviation
STAT: Signal transducer and activator of transcription
STING: Stimulator of interferon genes
TEAE: Treatment-emergent adverse event

## References

[1] Kim H, Sanchez GA, Goldbach-Mansky R (2016). Insights from Mendelian interferonopathies: comparison of CANDLE, SAVI with AGS. Monogenic Lupus J Mol Med (Berl).

[2] Liza NAS, Anwar SS, Kundu GK (2022). Aicardi-Goutieres syndrome-a case report. J Bangladesh CollPhys Surgeons.

[3] Arima K, Kinoshita A, Mishima H, Kanazawa N, Kaneko T, Mizushima T (2011). Proteasome assembly defect due to a proteasome subunit beta type 8 (PSMB8) mutation causes the autoinflammatory disorder, Nakajo-Nishimura syndrome. Proc Natl Acad Sci U S A.

[4] Brehm A, Liu Y, Sheikh A, Marrero B, Omoyinmi E, Zhou Q (2015). Additive loss-of-function proteasome subunit mutations in CANDLE/PRAAS patients promote type I IFN production. J Clin Invest.

[5] Liu Y, Ramot Y, Torrelo A, Paller AS, Si N, Babay S (2012). Mutations in proteasome subunit beta type 8 cause chronic atypical neutrophilic dermatosis with lipodystrophy and elevated temperature with evidence of genetic and phenotypic heterogeneity. Arthritis Rheum.

[6] Montealegre Sanchez GA, de Jesus AA, Goldbach-Mansky R, Orange JS, Chinen J (2020). Chronic atypical neutrophilic dermatosis with lipodystrophy and elevated temperature syndrome (CANDLE)/proteasome-associated autoinflammatory syndromes (PRAAS). Encyclopedia of medical immunology: immunodeficiency diseases.

[7] König N, Fiehn C, Wolf C, Schuster M, Cura Costa E, Tüngler V (2017). Familial chilblain lupus due to a gain-of-function mutation in STING. Ann Rheum Dis.

[8] Liu Y, Jesus AA, Marrero B, Yang D, Ramsey SE, Sanchez GAM (2014). Activated STING in a vascular and pulmonary syndrome. N Engl J Med.

[9] Crow YJ, Chase DS, Lowenstein Schmidt J, Szynkiewicz M, Forte GM, Gornall HL (2015). Characterization of human disease phenotypes associated with mutations in TREX1, RNASEH2A, RNASEH2B, RNASEH2C, SAMHD1, ADAR, and IFIH1. Am J Med Genet A.

[10] Orcesi S, La Piana R, Fazzi E (2009). Aicardi-Goutieres syndrome. Br Med Bull.

[11] Sanchez GAM, Reinhardt A, Ramsey S, Wittkowski H, Hashkes PJ, Berkun Y (2018). JAK1/2 inhibition with baricitinib in the treatment of autoinflammatory interferonopathies. J Clin Invest.

[12] Crow YJ, Vanderver A, Orcesi S, Kuijpers TW, Rice GI (2014). Therapies in Aicardi-Goutières syndrome. Clin Exp Immunol.

[13] Igaz P, Tóth S, Falus A (2001). Biological and clinical significance of the JAK-STAT pathway; lessons from knockout mice. Inflamm Res.

[14] Baricitnib FDA label. https://www.pmda.go.jp/files/000243207.pdf. Accessed 3 June 2022.

[15] Baricitinib Pharmaceutical and Medical Devices Agency. https://www.pmda.go.jp/files/000243207.pdf. Accessed 3 June 2022.

[16] Kim H, Brooks KM, Tang CC, Wakim P, Blake M, Brooks SR (2018). Pharmacokinetics, pharmacodynamics, and proposed dosing of the oral JAK1 and JAK2 inhibitor baricitinib in pediatric and young adult CANDLE and SAVI patients. Clin Pharmacol Ther.

[17] Vanderver A, Adang L, Gavazzi F, McDonald K, Helman G, Frank DB (2020). Janus Kinase Inhibition in the Aicardi-Goutières Syndrome. N Engl J Med.

[18] Filocamo G, Davi S, Pistorio A, Bertamino M, Ruperto N, Lattanzi B (2010). Evaluation of 21-numbered circle and 10-centimeter horizontal line visual analog scales for physician and parent subjective ratings in juvenile idiopathic arthritis. J Rheumatol.

[19] Mahoney FI, Barthel DW (1965). Functional evaluation: The Barthel index. Md State Med J.

[20] Argetsinger LS, Campbell GS, Yang X, Witthuhn BA, Silvennoinen O, Ihle JN (1993). Identification of JAK2 as a growth hormone receptor-associated tyrosine kinase. Cell.

[21] Keystone EC, Taylor PC, Drescher E, Schlichting DE, Beattie SD, Berclaz P-Y (2015). Safety and efficacy of baricitinib at 24 weeks in patients with rheumatoid arthritis who have had an inadequate response to methotrexate. Ann Rheum Dis.

[22] Gedik KC, Adeva GS, Wade J, Sanchez GM, de Jesus A, Goldbach-Mansky R, editors. Monitoring of BK Reactivation and Long-term Safety on JAK1/2 Inhibition with Baricitinib. Arthritis Rheumatol. Hoboken: Wiley; 2020.

