# Supplementary material for: Efficacy and safety of baricitinib in Japanese patients with autoinflammatory type I interferonopathies (NNS/CANDLE, SAVI, And AGS)
Source: Pediatr Rheumatol Online J. 2023 Apr 22;21:38. doi: 10.1186/s12969-023-00817-8 (PMC10122451; doi:10.1186/s12969-023-00817-8)
Supplement: Supplementary file 1 — Additional file 1: Supplementary Table S1. Daily symptoms of patients with NNS/CANDLE, SAVI, and AGS. Supplementary Table S2. Symptom-specific change from baseline in patients with NNS/CANDLE, SAVI, and AGS. [file 12969_2023_817_MOESM1_ESM.docx]

**Supplementary Table 1** Daily symptoms of patients with NNS/CANDLE, SAVI, and AGS

| **Disease State** | **Symptoms** | **Score Range** |
| --- | --- | --- |
| NNS/CANDLE | - Fever - Rash - Musculoskeletal pain - Headaches - Fatigue | - 0–4 - 0–4 - 0–4 - 0–4 - 0–4 |
| SAVI | - Fever - Rash - Musculoskeletal pain - Fatigue - Respiratory symptoms - Severity of ulcers/ischemic lesions | - 0–4 - 0–4 - 0–4 - 0–4 - 0–4 - 0–4 |
| AGS | - Neurologic disability - Crying - Length of uninterrupted sleep - Generalized seizure - Fever - Excessive irritability - Skin findings (body) - Skin findings (hands, feet, and ears) | - 0, 5, 7, 10 - 0, 1, 2, 3 - 0, 1, 2, 3 - 0, 8 - 0, 1 - 0, 1, 2, 3 - 0, 1, 2, 3 - 0, 1, 2, 3 |

AGS = Aicardi-Goutières syndrome; NNS/CANDLE = Nakajo-Nishimura syndrome/chronic atypical neutrophilic dermatosis with lipodystrophy and elevated temperature; SAVI = STING-associated vasculopathy with onset during infancy; STING = stimulator of interferon genes.

**Supplementary Table 2.** Symptom-specific change from baseline in patients with NNS/CANDLE, SAVI, and AGS

| **Disease** | **Symptom** | **N** | **Baseline** | **Primary Treatment (Week 20/32)** | | **Maintenance treatment (Week 52)** | |
| --- | --- | --- | --- | --- | --- | --- | --- |
|  |  |  |  | **Observed** | **Change** | **Observed** | **Change** |
|  |  |  | mean (SD) | mean (SD) | mean (SD) | mean (SD) | mean (SD) |
| NNS/CANDLE, mean (SD) | Fever | 5 | 0 (0) | 0 (0) | 0 (0) | 0 (0) | 0 (0) |
|  | Rash | 5 | 1.80 (0.84) | 1.20 (1.30) | -0.60 (0.90) | 1.31 (1.19) | -0.49 (0.87) |
|  | Headache | 5 | 0 (0) | 0.06 (0.13) | 0.06 (0.13) | 0.06 (0.13) | 0.06 (0.13) |
|  | Musculo-skeletal Pain | 5 | 1.69 (0.95) | 1.17 (0.85) | -0.51 (1.13) | 1.31 (1.06) | -0.37 (1.21) |
|  | Fatigue | 5 | 1.11 (1.09) | 1.09 (1.09) | -0.03 (1.0) | 1.03 (1.14) | -0.09 (1.17) |
| SAVI, mean (SD) | Fever | 3 | 0.10 (0.17) | 0 (0) | -0.10 (0.17) | 0 (0) | -0.10 (0.17) |
|  | Rash | 3 | 1 (0) | 0.67 (0.58) | -0.33 (0.58) | 0.67 (0.58) | -0.33 (0.58) |
|  | Musculo-skeletal Pain | 3 | 0.24 (0.41) | 0.05 (0.08) | -0.19 (0.33) | 0.05 (0.08) | -0.19 (0.33) |
|  | Fatigue | 3 | 1.38 (1.07) | 1.10 (0.86) | -0.29 (0.62) | 1.10 (0.86) | -0.29 (0.62) |
|  | Respiratory / Breathing Symptoms | 3 | 2 (1) | 1.62 (0.66) | -0.38 (0.54) | 1.29 (0.62) | -0.71 (1.12) |
|  | Ulcers / Ischemic Lesions | 3 | 0 (0) | 0 (0) | 0 (0) | 0 (0) | 0 (0) |
| AGS, mean (SD) | Neurologic Disability | 1 | 10 (-) | 10 (-) | 0 (-) | 10 (-) | 0 (-) |
|  | Crying | 1 | 0 (-) | 0 (-) | 0 (-) | 0 (-) | 0 (-) |
|  | Length of Uninterrupted Sleep | 1 | 0 (-) | 0.57 (-) | 0.57 (-) | 0.29 (-) | 0.29 (-) |
|  | Generalized Seizure | 1 | 0 (-) | 0 (-) | 0 (-) | 0 (-) | 0 (-) |
|  | Fever | 1 | 0 (-) | 0 (-) | 0 (-) | 0 (-) | 0 (-) |
|  | Excessive Irritability | 1 | 0 (-) | 0 (-) | 0 (-) | 0 (-) | 0 (-) |
|  | Skin Findings on Body | 1 | 0 (-) | 0 (-) | 0 (-) | 0 (-) | 0 (-) |
|  | Skin Findings, Hands, Feet, Ears | 1 | 0 (-) | 0 (-) | 0 (-) | 0 (-) | 0 (-) |

AGS = Aicardi-Goutières syndrome; NNS/CANDLE = Nakajo-Nishimura syndrome/chronic atypical neutrophilic dermatosis with lipodystrophy and elevated temperature; SAVI = STING-associated vasculopathy with onset during infancy; SD, standard deviation; STING = stimulator of interferon genes.
